# DIRECTIONS UNCLEAR: DEMYSTIFYING AGING AND FINDING OUR FUTURE GERONTOLOGISTS

**DOI:** 10.1093/geroni/igac059.1019

**Published:** 2022-12-20

**Authors:** Aaron Guest

**Affiliations:** Arizona State University, Phoenix, Arizona, United States

## Abstract

When did you first learn about gerontology? Becoming a gerontologist is rarely at the top of any student’s potential career list. How many of us only found the prospect of a gerontological career in graduate school – or after entering the workforce. We often hear that too few students show interest in jobs working with older persons. In turn, dedicated homes for gerontological degrees are threatened with closure and consolidation. Rather than students being uninterested, I believe the perceived ambiguity of careers and lack of exposure to aging content prevent many from seeking out these careers. In this lecture, I will share my own experience in navigating the ambiguity of a career in aging and how I now use it as a strength to recruit students. I aim to emphasize the unique role of gerontological education and the transdisciplinary approaches and interprofessional strengths that it provides for our aging society.

